# Letter to the Editor, “Treatment discontinuation in a post‐disaster outpatient clinic: A retrospective cohort study of post‐traumatic stress disorder and complex post‐traumatic stress disorder in Fukushima, Japan”

**DOI:** 10.1002/pcn5.70437

**Published:** 2026-11-03

**Authors:** Sarmad Ijaz, Anwar ul Haque, Amna Hassan Mohammed, Meer Hassan Khalid

**Affiliations:** ^1^ Shaikh Khalifa Bin Zayed Al‐Nahyan Medical And Dental College Lahore Pakistan; ^2^ Faculty of Medicine University of Khartoum Khartoum Sudan

We read with great interest the article by Hori et al., “Treatment discontinuation in a post‐disaster outpatient clinic: a retrospective cohort study of post‐traumatic stress disorder and complex post‐traumatic stress disorder in Fukushima, Japan,” published in *Psychiatry and Clinical Neurosciences Reports.* ^1^ In this single‐clinic study of 170 patients with ICD‐11 PTSD or complex PTSD, 33.5% discontinued treatment within 1 year, mostly within the first 90 days, and structured trauma‐focused psychotherapy reached only 17.6% of patients. The apparent protective association of psychotherapy disappeared when it was modeled as a time‐varying exposure (HR 0.482, 95% CI 0.113–2.050), leading the authors to conclude that early engagement is the critical bottleneck in PTSD care. We commend the authors for addressing treatment retention in a disaster‐affected population, an important and under‐researched topic, and for transparently reporting both the fixed‐exposure and time‐varying estimates. However, we would like to draw attention to the following limitations, which were not addressed by the authors.

First, the 1‐year outcome window is mismatched with the timing of psychotherapy initiation. The median time to structured psychotherapy was 327 days (IQR 78–629), so many patients started therapy near or after Day 365, and the article does not clarify how such exposure was coded in the time‐varying model. With only 28 exposed patients and 2 events, person‐time may be misattributed between exposed and unexposed states, which can bias the time‐varying HR (0.482) and weakens the claim that the crude association “largely reflected immortal time bias.” Lévesque et al. showed in a statin cohort that a strong protective association disappeared once immortal time was handled properly^2^. Second, remission, transfer, and death were censored rather than treated as competing events, and 26 patients were excluded from the analytic cohort based on their 1‐year outcome. Censoring assumes these events are non‐informative, which is unlikely because transfer and remission are clinically related to engagement. This may bias the continuation estimates and introduce selection bias. Austin et al. explain that censoring competing events overestimates the probability of the event of interest.^3^ Third, discontinuation was defined as cessation of visits, with no stated gap length or minimum number of visits. Patients who attended once and never returned are therefore pooled with those who dropped out of treatment, so the early peak in discontinuation may largely reflect non‐engagement. Szafranski et al. showed that PTSD dropout estimates depend heavily on how dropout is defined.^4^ Fourth, the 90‐day landmark is described as pre‐specified, yet it was chosen because 64.9% of events occurred before it, and testing of the proportional‐hazards assumption is not reported despite front‐loaded hazards. The finding that no variable predicted discontinuation after 90 days may therefore reflect the landmark choice and only 20 events. Dafni emphasizes that landmark times should be justified independently of outcome data and varied in sensitivity analyses.^5^ Finally, the conclusion that first‐90‐day interventions may hold greater potential than expanding psychotherapy capacity goes beyond the design, since no such intervention was evaluated, and the wide confidence interval is compatible with both benefit and harm. Hernán cautions against implying causal conclusions from observational data without an explicit causal framework.^6^ Other limitations, which we cannot discuss here for reasons of space, include the combined roles of a single clinician as diagnostician, therapist, outcome adjudicator, and analyst; heterogeneous and undocumented psychotherapy exposure; unreported handling of missing data and absence of sensitivity analyses; the absence of calendar‐time adjustment alongside an unsupported COVID‐19 attribution; and no stated adherence to STROBE or RECORD reporting guidelines.

To address these limitations, we recommend that the authors and future studies: (1) report exposed and unexposed person‐time, clarify how therapy starting after Day 365 was coded, and extend follow‐up; (2) analyze the full cohort using competing‐risk methods; (3) predefine a gap length and minimum visit threshold for discontinuation and test alternative definitions; (4) select landmark times a priori and report proportional‐hazards diagnostics; and (5) moderate causal conclusions until early‐engagement strategies are tested prospectively.

In conclusion, Hori et al.^1^ address an important, under‐researched problem, but their central claim that early engagement is the critical bottleneck rests on analyses with unclear handling of late‐starting exposure, competing events treated as censoring, a non‐standardized discontinuation definition, and an outcome‐driven landmark. Addressing these issues would strengthen this valuable work.

## AUTHOR CONTRIBUTIONS

**Sarmad Ijaz**: conceptualization; literature review; critical appraisal; manuscript drafting, manuscript revision. **Anwar ul Haque**: manuscript editing; critical revision. **Amna Hassan Mohammed**: conceptualization; manuscript review; final approval. **Meer Hassan Khalid**: literature review; supervision. All authors approved the final manuscript.

## CONFLICT OF INTEREST STATEMENT

The authors declare no conflicts of interest.

## ARTIFICIAL INTELLIGENCE (AI) DISCLOSURE

Artificial intelligence (AI)‐assisted writing tools were used solely to improve language clarity, grammar, and manuscript organization. The authors take full responsibility for the scientific content, interpretation, accuracy, and integrity of the manuscript.

## GUARANTOR

The corresponding author accepts full responsibility for the integrity and accuracy of the manuscript.

## PERMISSIONS

No copyrighted figures, tables, or unpublished materials requiring permission have been reproduced in this correspondence.

## ETHICS APPROVAL STATEMENT

As this manuscript is a correspondence/critical appraisal based exclusively on a previously published article, it did not involve human participants, patient data, animals, or identifiable information. Ethical approval was therefore not required.

## PATIENT CONSENT STATEMENT

Not applicable.

## CLINICAL TRIAL REGISTRATION

N/A.

## Data Availability

No new data sets were generated or analyzed during the preparation of this correspondence.
